# Teenage dogs? Evidence for adolescent-phase conflict behaviour and an association between attachment to humans and pubertal timing in the domestic dog

**DOI:** 10.1098/rsbl.2020.0097

**Published:** 2020-05-13

**Authors:** Lucy Asher, Gary C. W. England, Rebecca Sommerville, Naomi D. Harvey

**Affiliations:** 1 Centre for Behaviour and Evolution, School of Natural and Environmental Sciences, Newcastle University, Agriculture Building, Newcastle NE1 7RU, UK; 2 School of Veterinary Medicine and Science, University of Nottingham, Sutton Bonington Campus, Leicestershire LE12 5RD, UK; 3 The Royal (Dick) School of Veterinary Studies, University of Edinburgh, Easter Bush Campus, Bush Farm Road, Edinburgh, Midlothian EH25 9RG, UK; 4 Behaviour by Becca, Lexington Building, Bow Quarter, London E3 2UF, UK

**Keywords:** adolescence, dog, attachment, sensitive period, human–animal interaction, puberty

## Abstract

The relationship between parent and child changes around adolescence, with children believed to have: (i) an earlier puberty if they have less secure attachments to their carer; (ii) a phase of increased conflict behaviour toward their carer; and (iii) heightened conflict behaviour when carer attachments are less secure. We find support for analogous associations in adolescent dogs based on behaviour and reproductive timing of potential guide dogs. Bitches with behaviour indicative of insecure attachments pre-adolescence became reproductively capable earlier. Providing the first empirical evidence to our knowledge in support of adolescent-phase behaviour in dogs, we found a passing phase of carer-specific conflict-like behaviour during adolescence (reduced trainability and responsiveness to commands), an effect that was more pronounced in dogs with behaviour indicative of less secure attachments. These results indicate a possibility for cross-species influence on reproductive development and highlight adolescence as a vulnerable time for dog–owner relationships.

## 1. Introduction

Parent–child relationships share a surprising number of similarities with owner–dog relationships, including analogous behavioural and hormonal bonding mechanisms [1,2]. Adolescence is a vulnerable time for parent–child relationships, but little is known about owner–dog relations during adolescence. Adolescence is the final developmental stage of reproductive function, in which a juvenile becomes an adult, and incorporates puberty. In mammals, dramatic hormonal changes and reorganization of the brain [3,4] occur during puberty. When puberty starts so will potentially competing motivations in the domestic dog: to breed with conspecifics and to live in the care of humans. Together this means adolescence could be a vulnerable time for owner–dog relationships.

During puberty in humans, and alongside changes to hormones [5] and brain reorganization [6], there are transitory changes in risk taking, mood, irritability and conflict with parents (collectively known as ‘adolescent-phase behaviour’). Increased adolescent conflict behaviour between child and parent (generally mundane disagreements) is believed to be related to a need for individuation or autonomy [7,8]. Children with insecure attachments towards their carers are observed to have greater conflict and risk taking [9,10]. The timing of puberty is also associated with the quality of early relationships: children have an earlier onset of puberty if they have less attached, more insecure, relationships with carers [9,11–14].

Owing to behavioural and physiological similarities between parent–child and owner–dog relationships, the aim of this study was to examine the extent to which adolescence in dogs shares characteristics of adolescence in humans. Specifically, we investigated owner–dog parallels of three proposed characteristics of human parent–adolescent relations: (i) an earlier puberty for female dogs with less secure attachments to their carer; (ii) adolescent-phase conflict behaviour exhibited toward their carer; and (iii) greater conflict behaviour in dogs with less secure attachments to their carer.

## 2. Results

### (a) Influence of attachment on puberty

To investigate an association between attachment and puberty, we collected prospective data on attachment behaviour and monitored puberty (indicated by the first proestrus) in a cohort of 70 potential guide dog bitches born in 2012 (German shepherd dogs, golden and Labrador retrievers, and crosses of these). Attachment can be characterized by proximity seeking and distress upon separation [15] and relevant questions are found in two scales of the validated and widely used C-BARQ questionnaire [16], which we scored on a visual analogue scale (VAS). The first, Attachment and Attention Seeking was scored as a mean of six behaviours related to proximity seeking (e.g. ‘Tends to sit close to or in contact with you…’, ‘Displays a strong attachment for one… member of the household’) and the second, Separation-Related Behaviour, was scored as a mean of nine behaviours (e.g. ‘Shakes shivers of trembles when left, or about to be left’, ‘Appears agitated…when separated from you…’). We were able to confirm that these two scales were measuring insecure attachments, as higher scores in both scales were found in dogs categorized as insecurely attached based upon direct behaviour observations and using methods based on [17] (see Methods details in the electronic supplementary material). Since insecure attachments and pubertal timing could both be related to general fearfulness we also considered associations between puberty timing and a scale of general anxiety designed for this population [18]. Questions for these scales were completed by the dog's main carer, a Guide Dogs UK puppy walker whom the dog lives with from approximately 2 to 3 until 12 to 14 months of age.

**Figure 1.**
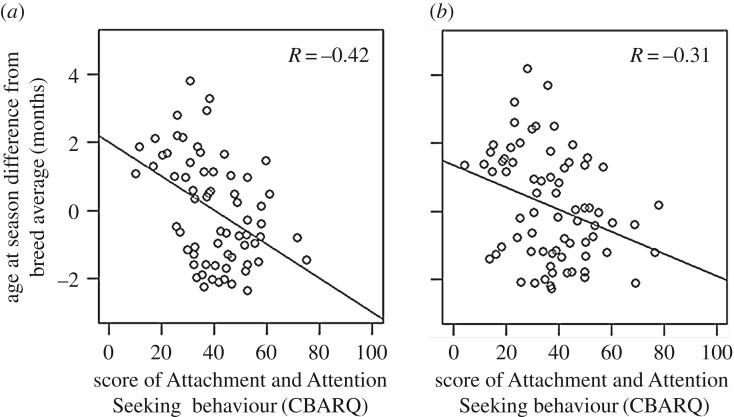
The negative association between insecure attachment behaviour measured by carers at 5 months and puberty end (first proestrus) relative to breed norm, based on: (*a*) partial correlation controlling for confounds of shared parentage and diet type; (*b*) correlation with no control for confounds. Attachment and Attention Seeking was scored on a 100 mm visual analogue scale, with a higher score indicating an insecure attachment.

Attachment and Attention Seeking was positively correlated with the age at which bitches had their first proestrus compared with their breed mean (calculated from population-level Guide Dogs UK records of all dogs born from 2012 to 2014). Bitches that displayed more Attachment and Attention Seeking behaviour at 5 months of age entered puberty earlier (figure 1*a*, *R* = −0.423, *n* = 64, *p* = 0.0004, based on partial correlation, controlling for diet and shared parentage confounds; and figure 1*b*, with no control for confounds, *R* = −0.315, *n* = 70, *p* = 0.007). Higher scores of Separation-Related Behaviour at 5 months of age were associated with entering puberty earlier when controlling for confounds (*R* = −0.295, *n* = 70, *p* = 0.014), but were not associated without control for confounds (*R* = −0.115, *n* = 70, *p* = 0.343). General anxiety was not associated with the timing of puberty without (*R* = −0.048, *n* = 70, *p* = 0.691) or with control for confounds (*R* = −0.134, *n* = 67, *p* = 0.271).

### (b) Adolescent-phase conflict behaviour

To investigate adolescent-phase conflict behaviour, we observed and scored obedience response of 93 dogs (41M: 52 F, breeds and cross breeds of: golden and Labrador retrievers) to an established command given by a carer and a consistent stranger in a controlled setting [19] (see Methods details in the electronic supplementary material). We predicted that dogs would be less obedient during adolescence, demonstrating an adolescent-phase of conflict with their primary carer. Reduced responsiveness to a well-established command (‘sit’) was considered as a proxy for reduced obedience. The population of dogs were sampled at pre-adolescent (*n* = 82 aged 5 months) and adolescent (*n* = 80 aged 8 months, of which 69 were tested at both time points) time periods. Dogs responded less to the ‘sit’ command during adolescence, but only when the command was given by their carer, not a stranger (the carer and stranger were the same people at both time points). The odds of repeatedly not responding to the ‘sit’ command were higher at 8 months compared with 5 months for the carer (odds ratio (OR) = 2.14, 95% confidence interval (CI) = 1.46–3.11, *Z* = 2.01, *p* = 0.044). However, the response to the ‘sit’ command improved for the stranger between the 5- and 8-month tests (OR = 0.40, 95% CI = 0.25–0.63, *Z* = 1.96, *p* = 0.049).

**Figure 2.**
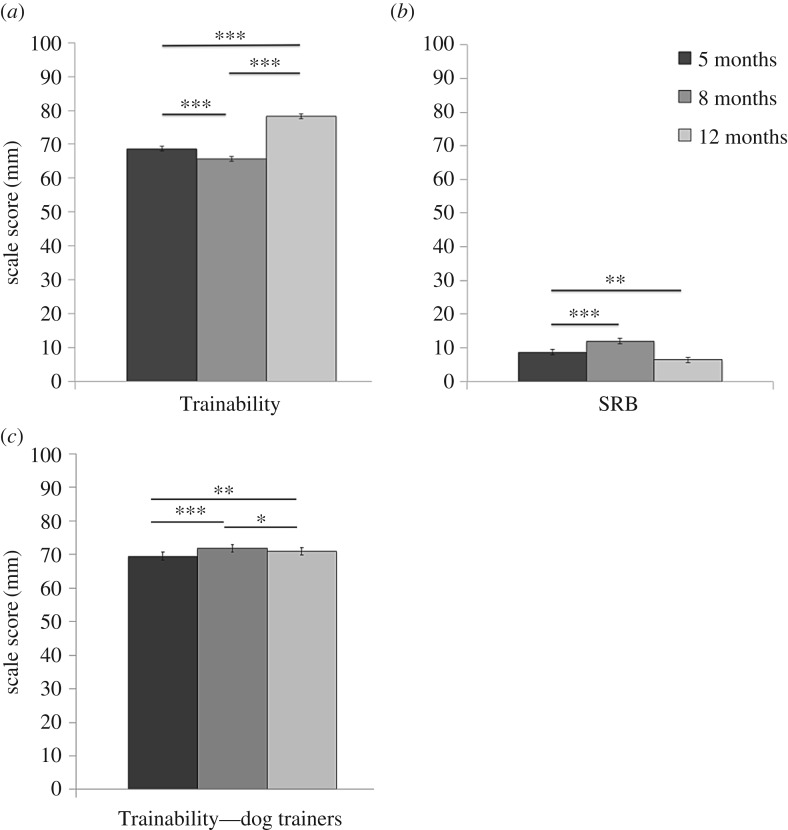
Scores for (*a*) Trainability (higher is more ‘trainable’) and (*b*) Separation-Related Behaviour (SRB, from C-BARQ where higher scores indicate more Separation-Related Behaviour displayed), as scored by dog carers (puppy walkers) when dogs were aged 5, 8 and 12 months. Scores for (*c*) Trainability when scored by the dogs' training supervisors when dogs were aged 5, 8 and 12 months. *p < 0.05, **p < 0.01, ***p < 0.001. Error bars represent s.e. of ±1.

Further evidence of a transitory adolescent-phase of disobedience confirming these findings was also found in data collected from a larger cohort of dogs (*n* = 285, 135 M : 150 F, breeds and cross breeds of: golden retriever, Labrador retriever and German shepherd dog) using the scale of ‘Trainability’ from two validated guide dog behaviour questionnaires completed by the dog's main carer [20], and a trainer (puppy training supervisor) less familiar to the dog [18]. Trainability was a mean of VAS scores to five questions (e.g. ‘This dog…refuses to obey commands, which in the past it was proven it has learned’, ‘Responds immediately to the recall command when off lead’). Carers assigned lower scores of Trainability to dogs around adolescence (8 months), than pre-adolescence (5 months of age) and post-adolescence (12 months). For carers, there was a 5- to 8-month decrease (cross-classified random effects GLM: *Z* = −4.46, *p* < 0.001) and a 5- to 12-month increase in Trainability on the questionnaire scale (*Z* = 13.76, *p* < 0.001, figure 2*a*). By contrast, the dog's trainers reported an increase in Trainability when adolescent (5- to 8-month increase: *Z* = 5.42, *p* < 0.001, figure 2*c*).

### (c) Adolescent-phase conflict behaviour and attachment

Questionnaires completed by dog carers were used to investigate whether adolescent conflict behaviour was associated with dog–carer attachment. Mirroring the transitory adolescent-phase of conflict was a phase of higher scores for Separation-Related Behaviour towards the carer. Scores for Separation-Related Behaviour were 36% higher at adolescence (8 months) than pre-adolescence (5 months) and post-adolescence (12 months) (5- to 8-month increase GLM: *Z* = 4.11, *p* < 0.001, and 5 to 12-month decrease GLM: 0.77, *Z* = 3.02, *p* < 0.01, figure 2*b*). Increased Separation-Related Behaviour at 8 months was associated with lower obedience (Trainability score) to their carer at 8 months of age (random effects GLM: *R* = −0.516, *t* = −10.37, *p* < 0.001), but not at 5 or 12 months. Scores of Attachment and Attention Seeking did not change with age, but they were correlated with Trainability at 8 months of age only (*R* = −0.298, *t* = −4.31, *p* < 0.001).

## 3. Discussion

The strength of attachment between humans and dogs is made possible by dogs piggybacking on human mechanisms for bonding with children [1,2]. Here, we find evidence to suggest that the human–dog attachment may in turn influence dog behaviour and reproductive physiology during puberty. Specifically, our results find an association between earlier puberty and an insecure attachment to a human carer. This replicates correlational findings from human adolescents who enter puberty earlier if they do not have strong attachments to parental figures [12]. Additionally, we found when dogs reached puberty, they were less likely to follow commands given by their carer, but not by others. The socially-specific nature of this behaviour in dogs (reduced obedience for their carer only) suggests this behaviour reflects more than just generalized hormonal, brain and reward pathway changes that happen during adolescence. In parts of this study, the ‘other’ person was a guide dog trainer who may have been more capable of getting a dog to perform a command; however, the results are consistent with parts of the study when the ‘other’ person was an experimenter without the experience of dog training. We also find the reduction in obedience to the carer and not an ‘other’ person to be specific to the dog's developmental stage and more pronounced in dogs with insecure attachments, which is not easily explained by differences in dog training ability between the carer and other.

We find support for the prediction that conflict behaviour is associated with less secure carer attachments during an adolescent-phase, because behaviour indicative of insecure or anxious attachments was only associated with obedience at an age that corresponds with adolescence. A weakness of this study is that puberty was not measured in all dogs, rather it was assumed based on existing knowledge of pubertal timing in relevant breeds (noting that age groupings would need to be reconsidered for different breeds). Further, when puberty was measured, our definition of the onset of puberty in females (first proestrus) was reductionist as some bitches may not have entered a complete cycle. We cannot preclude the possibility that the small minority of dogs were incorrectly classified as pubertal; however, this would be more likely to lead to a type II rather than type I error.

Research in rats and humans shows that adolescence is a sensitive period for development in mammals owing to the extensive reorganization of the brain's neural circuitry (see [21] for an overview). The possibility that puberty is a sensitive period in dogs warrants further investigation, particularly as experiences at this time could have long-term impacts on behaviour. A sensitive period around puberty is proposed in grey literature (e.g. within dog training literature); however, to our knowledge this is the first study to provide empirical support for this.

Reproductive development is known to be influenced by social relationships in a wide range of species [22], but this study highlights the possibility for cross-species influences on reproductive development. Like human adolescents, we find dogs' attachment behaviour to their carers is associated with the age at which puberty starts. It is likely that the carers' behaviour influences the dog's attachment to them [23]; indeed correlations have been found between human and dog attachments [24–26]. Understanding the specific behavioural influences on more secure attachments is an area for future study.

Our findings support dogs as a potential model species for studying puberty in humans. This is a particularly important area of study because early puberty is associated with more risky behaviour, earlier death, repeat offending, narcotics abuse and mental health problems [27]. Experimental studies of human puberty or attachment are not ethically possible but may be considered in dogs. Such studies could elucidate the causal link between attachment and pubertal timing, along with other aspects of adolescence. It will be important to confirm our results in future studies, because it is possible the similar results could arise from different explanatory mechanisms in dogs compared with humans.

We found that dogs displaying behaviour indicating they are stressed by separation from their main carer were also increasingly disobedient towards that same person. This finding emulates human research, where increases in conflict with parents during adolescence have been associated with insecure attachments [28]. An alternative explanation for our results is that some dogs received poorer training both in obedience and in being separated from their carer; however, our sample of trainee guide dogs were provided with standardized training to gradually introduce them to being left alone.

In humans, the conflict between parents and adolescents is proposed to function to test and potentially re-establish secure attachments [29]. A lack of secure attachments during childhood [30] and adolescence [31] is associated with earlier reproduction. In dogs, it is possible that the attachment to a carer acts as a cue of environmental quality, where the carer is the main source of survival. In this case, the attachment could have an evolutionary function to mediate between life-history strategies that favour roaming and early reproduction, versus continued human care and delayed reproduction.

In most dogs, it seems that adolescent-phase disobedient behaviour exists, but does not last. Unfortunately, the welfare consequences of adolescence-phase behaviour could be lasting because this corresponds with the peak age at which dogs are relinquished to shelters [32,33]. Welfare could be also be compromised if problem behaviour results in the use of punishment-based training methods [34] or causes carers to disengage, as it does in humans [35]. It is hoped these issues could be avoided if dog owners were made aware that (as in humans) problem behaviour during adolescence could be just a passing phase.

## Supplementary Material

Methods Electronic Supplementary Material

## Data accessibility

Currently provided as electronic supplementary material, but will be publicly available in the Newcastle University repository upon acceptance to allow tracking of downloads etc. Data are freely available in Dryad and can be accessed at doi:10.25405/data.ncl.12066270 [36].
