# Supplementary material for: Teenage dogs? Evidence for adolescent-phase conflict behaviour and an association between attachment to humans and pubertal timing in the domestic dog
Source: Biol Lett. 2020 May 13;16(5):20200097. doi: 10.1098/rsbl.2020.0097 (PMC7280042; doi:10.1098/rsbl.2020.0097)
Supplement: Methods Electronic Supplementary Material [file rsbl20200097supp1.docx]

Electronic Supplementary Material for: Teenage dogs? Evidence for adolescent-phase conflict behaviour and an association between attachment to humans and pubertal timing in the domestic dog

**Authors**: Lucy Asher, Gary C.W. England, Rebecca Sommerville, Naomi D. Harvey.

**Method Details**

**Experimental Model and Subject Details**

Overall, the subjects of this study were juvenile potential guide dogs born between May and July 2012. Caregivers of 276 of these dogs (130M: 146F) completed the Puppy Walker Questionnaire (PWQ) [1] including the separation relation behaviour (SRB) and attachment and attention seeking subscales of the Canine Behavior and Research Questionnaire (C-BARQ) [2]. These dogs included 8 different breeds or crossbreeds ([Sire x Dam]: Golden retriever x Labrador (n=105); Labrador (n=65), Golden retriever (n=30), Labrador x Golden retriever crossbreed (n=29), Golden retriever x German shepherd dog (n=24), German shepherd dog (n=16), Labrador x Golden retriever (n=5), and Labrador x Labrador crossbreed (n=2)). Differing subsets of these dogs were used for each analysis, depending on what additional data was available for them.

The ages at which dogs were evaluated by questionnaire in this study were 5, 8 and 12 months. Ages of 5, 8 and 12 months were selected to correspond to pre-adolescent, adolescent and post-adolescent points in the dog. Five months of age was selected because: male dogs are not fertile at 5 months; the inguinal rings which allow descent of the testis close by 6 months of age: and female dogs from the population studied were not fertile at this age (established using data on the earliest season of 984 bitches in the Guide Dogs UK population, which was 6.2 months). Eight months was selected because 95% of the 984 bitches had their first season between 6-12 months of age, and males are generally agreed to become fertile at around 6-12 months of age. Twelve months was selected because a large majority of dogs will have developed reproductive organs by this stage and dogs are commonly considered to be adults by 12 months of age.

The research adhered to legal and institutional ethical guidelines and received ethical approval from the School of Veterinary Medicine and Science’s ethics committee.

**Influence of attachment on puberty**

Since age of the final stage of reproductive organ maturity is visually apparent in female dogs, we used this property to measure the age at which dogs entered this final stage of puberty. The signs of proestrus include: a slight swelling of the dog's vulva, bright or dark red discharge from the dog's vulva an enlarged and turgid vulva. Caregivers were trained to notice these signs of proestrus. First proestrus was reported by dog’s caregivers, and confirmed by a guide dogs staff member who recorded this in their Guide Dogs health records. Guide Dogs staff and caregivers were not aware of the hypothesis being tested when data was collected. All female dogs were included if they had confirmed proestrus recorded, dogs with unconfirmed records were excluded.

Of the 146 females in the overall sample, confirmed data for the age at first proestrus was available for 70 bitches, and for 64 with information on potential confound of diet (nine different commercially available diets which cannot be revealed for commercially sensitive reasons) and size of dog (three weight categories of small: 21-27kg as adult; medium: 27-31kg; and large: 31+kg). Data is provided in Table S1.

**Adolescent-phase conflict behaviour**

Two datasets were used for the hypotheses relating to adolescent-phase conflict behaviour: 1) behaviour test responses from a subset of dogs testing in a juvenile guide dog behaviour test and 2) scores for all dogs on two behaviour questionnaires. Each data set is detailed below.

*Behaviour test responses*

A standardized juvenile guide dog behaviour test [3] was used to provide data on obedience and attachment at two age periods for a total of 93 dogs (41M: 52F, 38 Labrador retrievers and 47 crossbreeds between Labrador and Golden retrievers, 6 Labrador x F1 Golden retriever cross, and 1 German shepherd x Golden retriever). The dogs were first tested when they were pre-adolescent (mean age of 4.78 months ± 0.73 SD) and again when they would be expected to adolescent (mean age of 7.98 months ± 0.78 SD). In total 69 dogs were tested twice, 13 were tested only at pre-adolescence, and 11 were tested only at adolescence; there were no significant differences in breed or sex distribution at either test point.

Obedience tests were used to measure dog behaviour as a model for analogous to conflict behaviour with parents in human adolescents, which is characterized by minor mundane disagreements and a reduction in response to commands/requests. Obedience responses to an established command (sit) pre- and during adolescence. A reduced obedience response to a known command, “Sit” was used as a proxy for conflict behaviour and was tested as part of the juvenile guide dog behaviour test (subtests 2 & 3). During this test, dogs were given three commands in each subtest (sit, wait and down) firstly when handled by their caregiver (subtest 2) and secondly when handled by a stranger (subtest 3). The same female researcher acted as the ‘stranger’ in all tests. A test arena was demarked as an area 6.5m long and 4.5m wide using chairs. A pathway was marked in the test arena with markers on the floor to indicate where each command by the handler as they walked with the dog on a lead around the course. The markers were in three corners of the area. Cameras were placed in the middle of one width and one length of the area (Panasonic HDC-HS60 and wide-angle GoPro HD-Hero2). The number of commands required to elicit the desired response (sit, wait or down) was recorded to represent the dog’s obedience response from video footage. Intra-rater reliability was tested in 40 cases and was found to have an Intra class correlation coefficient of 0.93, which was deemed excellent. Only response to the ‘sit’ command was evaluated here as it was considered to be the only command that was fully established in all dogs during the first test when aged 5 months.

*Verifying measures of attachment*

Attachment type was measured using a modified version of the strange situation test with observations of behaviour during interactions, separation and reunion from a stranger and the main caregiver at 5 and 8 months of age. The test was modified as it occurred as part of a wider battery of behaviour observations. When other family members brought the dog to the test, only the main caregiver (puppy walker) continued into the test room to participate in this section. Before testing began the dog was offered water and a short off-lead break. The stranger was always female and unknown to the dog. There were three strangers across the study in total, and consistency in approach between testers was maintained by extensive briefing, detailed protocols and watching previous videos. Observations were made of separation and reunion behaviour toward their caregiver and the stranger when the caregiver and stranger was present and then left the room during engagement in play (first unstructured, then structured). During the unstructured play the caregiver was given instructions to play with the dog as they normally would at home until the stranger returned. A large box with a range of toys was provided with five soft toys, two soft and two rubber squeaking toys, two rubber kongs, two rubber rings and three soft-rope toy combinations. The tester left the room for 90 seconds before returning to the room and returning toys to the box. During the structured play a toy (‘Ruff and Tuff’ medium knotted rope, Pets at Home Ltd.) was thrown from a standing marker to another marker approximately 2m distance and the dog was encouraged to engage with the toy and return it to the person. The is repeated two times and then the dog was encouraged to engage in tug with the person. This was repeated with both the caregiver or stranger (in a counter-balanced order) with the other person quietly exiting the room and returning after 60 seconds. Using video footage from cameras described as in [3], the following behaviour was measured during the different phases using continuous sampling: Looked at exiting or returning person, Ran or walked to person, Jumped up at person, Scratched door, Waited by door (>3s), Touched person, Vocalised, Followed person through door, No reaction; engaged in play behaviour. This behaviour was scored blind to the hypothesis and inter and intra-rater reliability was assessed by scoring videos twice, six weeks apart. Cohens alpha was used to assess rater reliability and above 0.69 in all cases. This was deemed acceptable especially since some behaviours were rare. The behaviour data allowed dogs to be categorised according to methods presented in [4] noting that these classifications have an emphasis on behaviour during reunion, rather than the behaviour towards the stranger in the presence or absence of the caregiver. Using this approach we were able to classify 47 dogs as either: Attached and Secure (Secure in original paper); Attached and Insecure (Insecure-Ambivalent); and Avoidant. No cases fitted with descriptions of Insecure-disorganized/disoriented, and these dogs may have been grouped with unclassified.

Researchers conducting the test and caregivers were blind to the hypotheses being tested. All juvenile potential guide dogs born between May and July 2012 were invited to take part in the study and were only excluded based on caregivers’ availability to attend the testing. Data is provided in Table S2.

*Questionnaire data*

Sub scales from three questionnaires were used to collect the data for this study. Two sub-scales from the widely validated C-BARQ [2], Attachment and Attention Seeking and Separation-related behaviour, were used. The C-BARQ is typically scored using a five-point Likert scale, however to increase discriminatory power, to avoid statistical bias and influence of respondent style, we used a Visual Analogue Scale. The suitability of using a VAS instead of a Likert scale for these C-BARQ scales had been established in a previous publication [1]. The Attachment and Attention Seeking scale was comprised of six questions on “Tends to follow you (or other member of household) about the house from room to room”; “Tends to sit close to or in contact with you (or others) when you are sitting down”; “Tends to nudge, nuzzle, or paw you (or others) for attention when you are sitting down”; “Becomes agitated (whines, jumps up, tries to intervene) when you (or others) show affection for another person”; “Becomes agitated (whines, jumps up, tries to intervene) when you show affection for another dog or animal”; “Displays a strong attachment for one particular member of the household”. The Separation-related behaviour scale is comprised of nine questions: “Shakes shivers of trembles when left, or about to be left”; “Salivates excessively when left, or about to be left; “Appears restless/agitated or paces when left, or about to be left”; “Whines when left, or about to be left”; “Barks when left, or about to be left”; “Howls when left, or about to be left”; “Chews/scratches at doors, floor, windows, curtains etc. when left, or about to be left”; “Loses its appetite when left, or about to be left”; “Appears agitated (whines, barks, howls, scratches at door etc.) when separated from you (or a member of the household) but not alone”. Each question was scored on a visual analogue scale (VAS) which was 100mm in length with the anchors “Never” to “Almost Always”. The final score was a mean of all questions from 0-100. Caregivers who completed the questionnaire were not aware of the hypothesis being tested when data was collected.

General Anxiety was evaluated by a scale composed of five questions. These questions are proceeded by “This dog…”: “Is obviously disturbed by loud or unexpected sounds”, “Is spooked by odd or unexpected things or objects”, “Is anxious or uneasy in new situations, “Backs away from or is reluctant to pass objects on the street (such as collecting boxes, bin bags or children's ride-on toys)”. This is similar to the C-BARQ non-social fear, but was designed and validated for use in this distinct population of trainee guide dogs (for more details see [1,5]).

Trainability was evaluated by a scale composed of five questions. These questions are proceeded by “This dog…”: “Seems not to listen even when it knows someone is speaking to it”; “Refuses to obey commands, which in the past it was proven it has learned”; “Needs obedience commands repeating to get a response”; “Stays/waits when instructed to”, “Responds immediately to the recall command when off lead”. Each question was scored on a visual analogue scale which was 100mm in length from “Never” to “Almost Always” (anchors). The final score was a mean of all questions from 0-100, following reversal of the three negative (disobedience) questions, such that higher scores indicated greater ‘Trainability’. The Trainability scale was scored by the dogs puppy walkers via the Puppy Walker Questionnaire [1] at the same time as they scored the C-BARQ scales. A Guide Dogs’ UK member of staff, the ‘Puppy Training Supervisor’ for each dog also completed scores of Trainability at the same time using the Puppy Training Supervisor Questionnaire (PTSQ) [6]. The Trainability data from the questionnaires was used to confirm results from the obedience test and distinguish differences in obedience behaviour towards their main caregiver (in the PWQ) and a less familiar handler (in the PTSQ). All caregivers (Puppy walkers) of juvenile potential guide dogs born between May and July 2012 (n=311) were invited to participate in this study and response rate was 61%. Data is provided in Table S3.

**Quantification and statistical analysis**

***Verifying measures of attachment***

Associations were tested between scores of Attachment and Attention Seeking and Separation Related Behaviour subscales from C-BARQ and independent data on attachment category based on direct behaviour observations. Data were analysed using linear mixed model fitted in R (version. 3.4.1, R core team, 2017 [7]), where C-BARQ scale was the dependent variable, Attachment category, Assessment point (5, 8 or 12 months), and the interaction between these were fixed effects, and DogID was a random effect. Models were simplified by removing variables and interactions which did not alter model fit (assessed by anova() command to compare models in R). Results are presented here on the relationship between subscales and attachment category as they provide methodological support for use of these scales in subsequent analyses.

Attachment and attention seeking scores from the C-BARQ were associated with insecure attachments, with dogs scoring lower on this scale if they were categorised as Avoidant (by -12.85 ± 4.88 F_2 42.43_= -2.64, p= 0.018) or Attached and Secure (by -15.74 ± 4.97 F_2 42.43_= -3.166, p= 0.002). Separation related behaviour scores on C-BARQ were also associated with Insecure attachments compared to Attached and Secure (by -5.87 ± 2.84 F_2 17.22_= -2.07, p= 0.042). There was also a trend for Avoidant dogs to be scored lower on this scale than insecurely attached (by -5.17 ± 2.82 F_2 17.22_= -1.84, p= 0.072). If this category is analogous to human infants then despite few outwards signs of separation distress, avoidant dogs may experience stress upon separation, thus such dogs may be more likely to develop separation related behaviour problems, than securely attached dogs. These findings were deemed to provide sufficient evidence for the two subscales to be used as measures or proxy measures of an insecure attachment in further analyses.

***Testing prediction i): later puberty onset for dogs with more secure caregiver attachment.***

Scores for the C-BARQ (Canine Behavioral Assessment and Research Questionnaire) Attachment and attention seeking (AAS), Separation-related behaviour (SRB) from the C-BARQ and a General Anxiety scale designed for juvenile guide dogs completed by main care givers at five months of age were compared to age at proestrus, whilst controlling for diet and litter. Diet has an association with reproduction (internal Guide Dogs data not shown) and Litter controls for genetic and nest environment variance shared between siblings from the same litter. Size was also initially included as a predictor but was dropped as it explained little variance and was correlated with diet. Data were analysed via a cross-classified linear mixed model fitted in R (version. 3.4.1, R core team, 2017 [7]. The model can be written as:

AgeAtProestrus_i_ = β 0iconsi + β1AAS

[u(3)0,Litter_ID(i)] ~ N(0, Ω(3)u) : Ω(3)u = [Ω(3)u0,0]

[u(2)0,Diet(i)] ~ N(0, Ω(2)u) : Ω(2)u = [Ω(2) u0,0]

[e 0i] ~ N(0, Ωe) : Ωe = [Ωe0,0]

Where β 0i represents the intercept of the model and the mean age at first proestrus. β1 represents the coefficient for AAS.

Random effects terms were then removed and a linear model was used to analyse the association between age at proestrus and the three scales from the C-BARQ completed by main care givers at five months of age. Model diagnostics and assumptions were checked using plot(*model*) and qqnorm() functions in R. Association between Age at proestrus and AAS/SRB/General Anxiety are reported in the paper as R values, produced based on the model with random effects (partial correlation), and the same model with random effects terms removed (correlation). The effective sample size for each model, n and the P value are also reported.

***Testing prediction ii): dogs exhibit a transitory adolescent phase of conflict behaviour toward the caregiver*.**

Decreases in responses to the known command “Sit” were considered to indicate conflict with differences between handlers, the caregiver or a stranger, used to interpret whether changes in obedience were generalized or specific to the caregiver. Cumulative Link Mixed Model fitted with the Laplace Approximation was used to compare the dog’s response to the ‘Sit’ command between handlers at each age (5 months and 8 months) towards a caregiver and stranger (part of the juvenile guide dog behaviour test, 16). ‘Sit’ response was recorded as an ordinal outcome, with a 1 indicating immediate response to command, 2 indicating a response after two or more commands, and 3 indicates no response to command. The ‘Sit’ response (1, 2 or 3) was the outcome variable with fixed effects of age (5 or 8 months) and handler (caregiver or stranger) and a random effect of Dog ID. The model was fitted in R using the clmm command in the package ordinal and goodness of fit of this model was checked using the rms.gof package.

The model can be written as:

logit(P(Siti ≤ j)) = θ j - β1Stranger_5M i – β2Caregiver_8M i +β3Stranger_8M- u(DogID i)

We assume dog effects to be random and normally distributed:

[u (DogID(i))] ∼ N(0, Ωu)= [Ωu0,0].

Where i = index of observations, 1, . . .n, j = response categories, 1, 2,3. θ j is the threshold parameters (logit) intercepts for each category of j (Sit response) for the comparator of 5 month old dogs handled by their caregiver, β1 represents the coefficient for dogs handled by a stranger at age 5 months, β2 for dogs handled by their caregiver at age 8 months and β3 for dogs handled by a stranger at age 8 months. The odds ratio of being in a higher (worse) category of “Sit” response the 95% Confidence Interval of this, Z and P-value are reported for each significant coefficient (P<0.05).

Trainability scores in a larger cohort of dogs were used to further test the prediction that obedience would reduce around the age of adolescence. Cross-classified multi-level models were utilised to identify patterns of change with age in the scores for the traits Trainability from the Puppy Walker Questionnaire (PWQ) and Puppy Training Supervisor Questionnaire (PTSQ) scored at 5, 8 and 12 months, whilst controlling for effects of litter (sibling that shared a nest), sire, dam and supervisor (the staff member responsible for supporting training and care of the puppy). Models were computed using MLwIN v.2.26 (Centre for Multilevel Modelling, University of Bristol) with Markov Chain Monte Carlo (MCMC) methods used for parameter estimation. MLwIN was needed for this analysis due to the structure of variance from the random effects. The model equation for Trainability as scored on the PWQ and PTSQ can be written with classification notation as:

Trainability = β 0iconsi + β1Age_8M + β2Age_12M

β 0i = β 0 + u(5)0,PTS_ID(i) + u(4)0,Sire_ID(i) + u(3)0,Dam_ID(i) + u(2)0,Dog_ID(i) + e0i

[u(5)0,PTS_ID(i)] ~ N(0, Ω(5)u) : Ω(5)u = [Ω(5)u0,0]

[u(4)0,Sire_ID(i)] ~ N(0, Ω(4)u) : Ω(4)u = [Ω(4)u0,0]

[u(3)0,Litter_ID(i)] ~ N(0, Ω(3)u) : Ω(3)u = [Ω(3)u0,0]

[u(2)0,Dog_ID(i)] ~ N(0, Ω(2)u) : Ω(2)u = [Ω(2) u0,0]

[e 0i] ~ N(0, Ωe) : Ωe = [Ωe0,0]

Where β 0i represents the intercept of the model and the mean for a dog at five months of age. β1 and β2 represent coefficients for age of assessment. The variance associated with the random effects is represented by the following terms for the supervisor variance:

[u(5) 0,PTS_ID(i)] ~ N(0, Ω(5) u) : Ω(5) u = [Ω(5) u0,0]

To the residual variance:

[e 0i] ~ N(0, Ωe) : Ωe = [Ωe0,0]

Model outputs were assessed for estimates of reliability in relation to chain length using MCMC diagnostics. The Raftery-Lewis diagnostic was used to estimate the chain length required to accurately estimate the 2.5% and 97.5% quartiles, and the Brooks-Draper diagnostic was used to estimate the chain length required to accurately estimate the mean [8]. All models were run to a chain length sufficient to meet these requirements for the model parameters. In addition to this, thinning (a process which dictates the frequency of storing successive values in the Markov chain) of 25 or 50 was used where required to reduce auto-correlations [9]. Parameter estimates, Z values and P values are reported for significant results where significance is considered to be P<0.05.

***Testing prediction iii):* reduced relationship security in dogs exhibiting greater conflict**

Separation-Related Behaviour was used as an indicator of reduced dog-owner relationship security, and lower Trainability scores as an indicator of conflict. Cross-classified multi-level models (as above in MLwIN) were utilised to identify patterns of change with age in the scores for Separation-Related Behaviour from the puppy walker questionnaire (PWQ) scored at 5, 8 and 12 months, whilst controlling for effects of litter (sibling that shared a nest), sire, dam and supervisor (the staff member responsible for supporting training and care of the puppy). Parameter estimates, Z values and P values are reported for significant results where significance is considered to be P<0.05. Finally, a linear mixed model implemented in R was used to test for associations between Separation-Related Behaviour and Trainability (both from PWQ) at each age of testing. Trainability was the outcome variable, Separation-Related Behaviour was a covariate which was stratified by Age (5, 8 or 12 months), and DogID was included as a random effect term. The model was used without assumptions about the causality of association between Separation-Related Behaviour and Trainability, but rather to extract correlations at each age whilst controlling for random effects. Model diagnostics and assumptions were checked using plot(model) and qqnorm() functions in R. Parameter estimates and standard error of estimates, t values and P values are reported for significant results where significance is considered to be P<0.05.

Supplementary Open Data

**Supplementary Table S1.** Age of first season data for 70 dogs used in this study.

**Supplementary Table S2.** Data for 'sit' performance in the juvenile guide dog behaviour test; 1 indicates immediate response to command, 2 indicates a response after two or more commands, and 3 indicates no response to command. PW = puppy walker (caregiver), STR = stranger.

**Supplementary Table S3**. Repeated measures data from the puppy walking questionnaire scale Trainability and C-BARQ scale separation-related behaviour (SRB) used in multi-level and mixed modelling. Anonymous ID codes have been assigned to each dog, dam, sire and supervisor. Assessment point indicates the dog’s age in months at which the questionnaire was completed.

References

1. Harvey ND, Craigon PJ, Blythe SA, England GCW, Asher L. 2016 Social rearing environment influences dog behavioral development. *J. Vet. Behav. Clin. Appl. Res.* **16**, 13–21. (doi:10.1016/j.jveb.2016.03.004)

2. Hsu Y, Serpell JA. 2003 Development and validation of a questionnaire for measuring behavior and temperament traits in pet dogs. *J. Am. Vet. Med. Assoc.* **223**, 1293–1300. (doi:10.2460/javma.2003.223.1293)

3. Harvey ND, Craigon PJ, Sommerville R, McMillan C, Green M, England GCW, Asher L. 2016 Test-retest reliability and predictive validity of a juvenile guide dog behavior test. *J. Vet. Behav. Clin. Appl. Res.* **11**, 65–76. (doi:10.1016/j.jveb.2015.09.005)

4. Solomon J, Beetz A, Schöberl I, Gee N, Kotrschal K. 2019 Attachment security in companion dogs: adaptation of Ainsworth’s strange situation and classification procedures to dogs and their human caregivers. *Attach. Hum. Dev.* **21**, 389–417. (doi:10.1080/14616734.2018.1517812)

5. Harvey ND. 2014 Development and Evaluation of Methods for Determining Personality in Juvenille Guide Dogs. University of Nottingham.

6. Harvey ND, Craigon PJ, Blythe SA, England GCW, Asher L. 2017 An evidence-based decision assistance model for predicting training outcome in juvenile guide dogs. *PLoS One* **12**, e0174261. (doi:10.1371/journal.pone.0174261)

7. Team R core. 2017 R: a language and environment for statistical computing.

8. Fielding A, Goldstein H. 2006 Cross-classified and multiple membership structures in multilevel models: An introduction and review.

9. Browne WJ. 2012 *MCMC estimation in MLwiN Version 2.26*. Bristol, United Kingdom: Centre for Multilevel Modelling University of Bristol.
